# Real-world application of an eye-tracking device for autism screening and diagnosis: a short report from public demonstrations in Qatar, Dubai and the U.S

**DOI:** 10.1186/s12888-026-07840-5

**Published:** 2026-01-30

**Authors:** Fouad Al Shaban, Thomas W. Frazier, Iman Ghazal, Fatema Al-Faraj, Sarah Aqel, I. Richard Thompson

**Affiliations:** 1 Neurological Disorders Research Center, Qatar Biomedical Research Institute, Hamad Bin Khalifa University, Qatar Foundation, Doha, Qatar; 2 College of Health and Life Sciences, Hamad Bin Khalifa University, Qatar Foundation, Doha, Qatar; 3 Department of Psychology, John Carroll University, Cleveland, OH USA; 4 Department of Pediatrics and Psychiatry, SUNY Upstate Medical University, Syracuse, NY USA; 5 Autism Speaks, New York, NY USA; 6 North East and Yorkshire NHS Genomic Laboratory Hub, Leeds, England

**Keywords:** Autism spectrum disorder, Eye-tracking, Autism index, Social attention, Screening tool, Public demonstrations, Diagnostic accuracy

## Abstract

**Background:**

Social attention abnormalities are a hallmark of Autism Spectrum Disorder (ASD), often characterized by atypical gaze patterns.

**Aim:**

This report showed the real-world feasibility of an eye-tracking–based screening paradigm for ASD across diverse general population samples.

**Methods:**

A total of 536 adults participated in public demonstrations. An Autism Index (AI), derived from a previously validated eye-tracking paradigm, was calculated from gaze patterns toward social and nonsocial stimuli. Participants were stratified into typically developing (TD), neurodivergent, and ASD groups based on validated AI cut-offs.

**Results:**

Median AI scores differed significantly across groups − 0.31 (TD), 0.53 (neurodivergent), and 0.69 (ASD)- with post-hoc tests confirming higher scores in the ASD and neurodivergent groups versus TD. Gender-based analyses showed that males had significantly higher AI scores than females (*p* = 0.028). Among those classified as ASD, 95% reported a formal diagnosis, supporting the validity of the tool. Receiver operating characteristic (ROC) analysis demonstrated excellent diagnostic performance, with an area under the curve (AUC) of 0.997. At the optimal cut-off score of 0.555, the tool achieved 100% sensitivity and 99.4% specificity.

**Conclusion:**

This report highlights the feasibility and accuracy of the eye-tracking paradigm as a scalable and objective screening tool for ASD in general adult populations, supporting its potential for broader clinical implementation.

**Clinical trial number:**

Not applicable.

## Background

Social attention, a fundamental aspect influencing developmental processes such as social cognition, has consistently shown abnormalities in individuals with autism spectrum disorder (ASD) [1–6]. Numerous studies have identified characteristic differences in gaze patterns between autistic individuals and controls, notably reduced attention toward social stimuli and increased preference for nonsocial stimuli [5, 7–9]. These gaze differences emerge early in development [10] and persist into adulthood, representing a reliable behavioral feature of ASD. Indeed, atypical gaze patterns toward social cues have been considered diagnostic hallmarks and remain critical components in current diagnostic tools [11, 12]. Despite the utility of established diagnostic measures, these clinical tools rely heavily on subjective evaluations, which can be prone to variability and require extensive training to achieve reliability [13–15]. Consequently, there is a growing emphasis on developing objective measures for ASD screening, such as eye-tracking technology, which provides quantifiable, objective data through the application of gaze-based metrics during viewing of socially-relevant stimuli [16]. Recent studies have demonstrated that gaze data from social and nonsocial areas-of-interest within socially-relevant stimuli can be aggregated into objective indices that effectively differentiate individuals with ASD from non-autistic controls, suggesting their potential for cross-cultural adaptation [3, 17–21].

Recently, our research group developed and validated an eye-tracking paradigm for the objective of screening and diagnosis of ASD, demonstrating strong reliability and validity [22]. This report outlines our experience using the paradigm at international conferences attended by a diverse audience. We aim to test the real-world application and effectiveness of this validated eye-tracking device when used within the general adult population.

## Methods

### Setting and data collection

Data were collected during three major international events— Arab Health, Dubai 2025 (www.arabhealthonline.com), Web Summit Qatar, 2025 (www.websummit.com) and INSAR 2025 Annual Meeting, Seattle, Washington, USA (www.autism-insar.org/page/2025AnnMeeting). These conferences attract large number of attendees from diverse professional backgrounds, including healthcare, technology, and innovation, rather than specifically targeting ASD-related fields. Visitors to our booth voluntarily engaged with the eye-tracking device out of personal interest, making the voluntary response sample more consistent with a general population adult audience than a referred clinical sample. The data were collected for descriptive purposes, such as assessing device engagement, usability, and performance in a non-clinical setting. All data were collected anonymously and used solely for the purpose of evaluating system performance in real-world conditions. Participants were not actively recruited; instead, they voluntarily took part in a public demonstration of the eye-tracking device during the events. At the time of testing, there was no intent to use the findings for research or publication. However, the data were later identified as having potential scientific relevance and were analyzed in fully de-identified and aggregated form. No additional data were collected, and no re-contact with participants occurred. Due to the public, non-research nature of these demonstrations, individual informed consent was not obtained or required. No confidential or identifiable data was collected during these demonstrations. The eye-tracking research protocol underlying the technology was approved by the Qatar Biomedical Research Institute Institutional Review Board (QBRI-IRB-2023-40; March 2, 2023). The authors assert that all procedures contributing to this work comply with the ethical standards of the relevant national and institutional committees on human experimentation and with the Helsinki Declaration of 1975.

### Eye-tracking paradigm and autism risk index (AI)

The Autism Index (AI) used in this study was calculated by aggregating eye-tracking metrics of gaze patterns toward social and nonsocial stimuli. Specifically, the AI integrates gaze indicators reflecting reduced attention toward social cues and increased attention toward nonsocial elements, generating a continuous score that quantifies a social attention gradient [3]. Higher AI scores indicate a greater likelihood of ASD. The eye-tracking device used was the SMI RED250 remote eye-tracker system, which is attached to a 19-inch LCD monitor used for stimulus presentation. This system captures binocular gaze, three-dimensional eye position, pupil dilation, and timestamp data at a frequency of 250 Hz. The setup included a calibration procedure to ensure accuracy, achieving maximum precision within 0.4 degrees. Participants viewed stimuli consisting of dynamic individual faces, social interactions, geometric patterns, and static face presentations. Detailed acquisition methods and analyses of these metrics are described extensively in the validation paper by Al-Shaban et al. [22]​.

### Participants group classification

Participants were categorized into three groups—typically developing (TD), neurodivergent, and autism spectrum disorder (ASD)—based on their Autism Index (AI) scores. The cut-off thresholds used to define these groups were originally derived from clinical data in our prior validation study conducted in Qatar by Al-Shaban et al. (2023) [22], where AI scores were benchmarked against gold-standard diagnostic assessments such as the ADOS-2 and the SCQ. These clinically established thresholds were applied to the current general population sample to enable consistent group stratification. Participants with AI scores between 0.00 and 0.50, reflecting typical social attention patterns, were classified as typically developing. Those with AI scores between 0.51 and 0.55, reflecting gaze patterns falling between the TD and ASD thresholds, were classified as neurodivergent. Participants with AI scores of 0.56 or higher, characterized by gaze patterns associated with ASD, were classified as ASD. All participants, regardless of AI-based classification, were asked privately whether they had a prior formal diagnosis of ASD or had previously been advised by a healthcare professional to seek assessment for autism-related features.

### Statistical analysis

We first assessed the normality of the Autism Index (AI) scores using skewness and kurtosis values. As the AI scores were not normally distributed, descriptive statistics were reported using the median and interquartile range (IQR), and non-parametric tests were employed for group comparisons. The Kruskal-Wallis test was used to assess differences in AI scores across the three groups (TD, neurodivergent, and ASD), followed by Bonferroni-adjusted post-hoc tests for pairwise comparisons. The Mann-Whitney U test was used to evaluate gender-based differences in AI scores. Receiver operating characteristic (ROC) curve analysis was performed to assess the diagnostic accuracy of the AI scores in distinguishing ASD from non-ASD participants, using self-reported prior clinical ASD diagnosis as the reference standard. The optimal cut-off was determined using Youden’s Index. False negatives were defined as participants with a reported ASD diagnosis whose AI score fell below the selected cut-off (*n* = 0), while false positives were defined as participants without a reported ASD diagnosis whose AI score exceeded the cut-off (*n* = 5). Sensitivity, specificity, and likelihood ratios were calculated. All statistical tests were two-tailed, with significance set at *p* < 0.05.

## Results

**Fig. 1:**
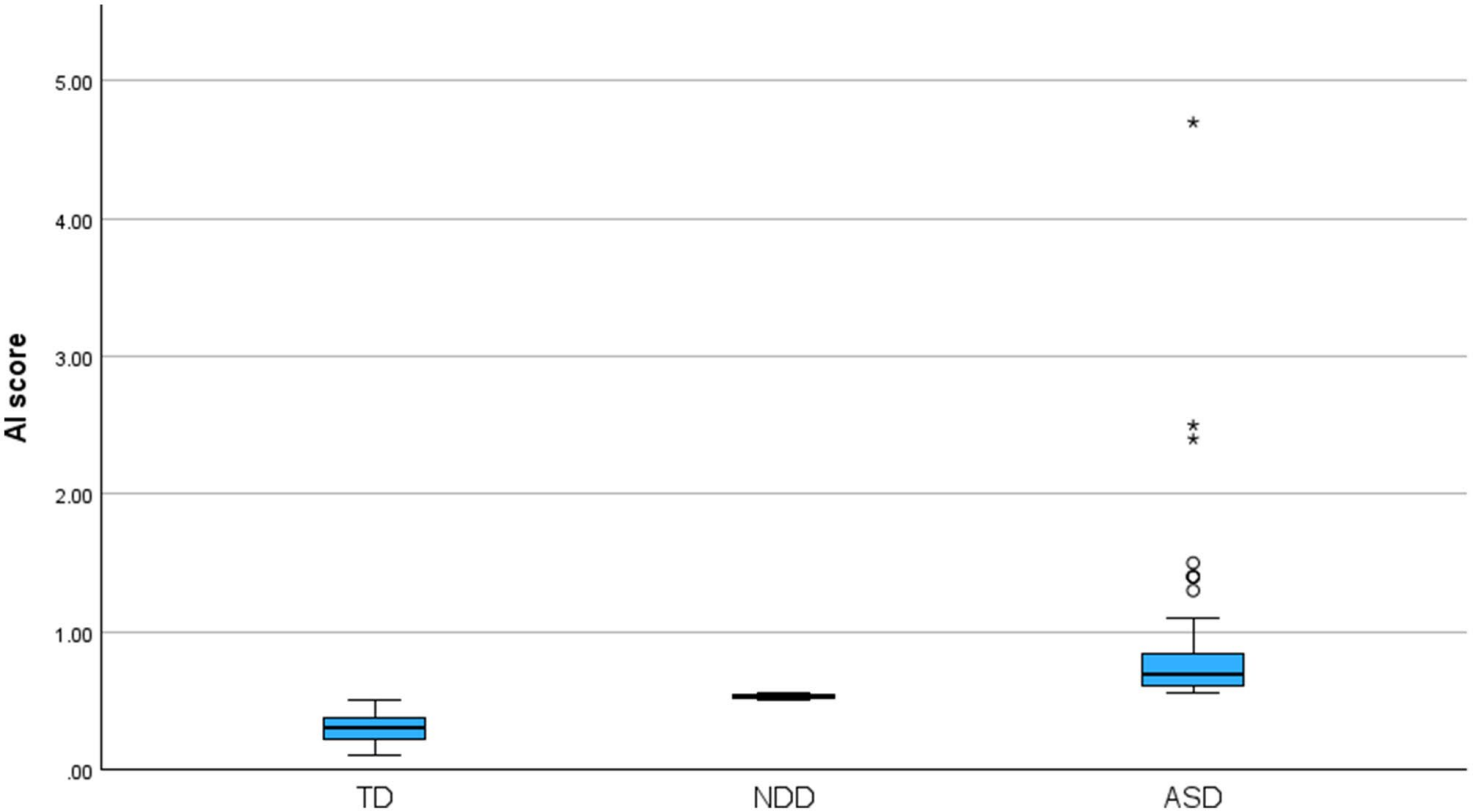
Distribution of AI scores across TD, neurodivergent, and ASD groups

A total of 536 adult participants took part in the eye-tracking demonstrations, with 48.2% (*n* = 260) identifying as male and 51.2% (*n* = 276) as female, resulting in a male-to-female ratio of approximately 0.94:1. Assessment of normality (skewness = 7.397, kurtosis = 89.442) ) indicated that AI scores were not normally distributed. AI scores ranged from 0.11 to 4.70, with a median score of 0.34 (IQR: 0.20). Participants were classified into three groups based on AI thresholds: typically developing (TD, *n* = 446, 82.7%), neurodivergent (*n* = 22, 4.1%), and ASD-positive (ASD, *n* = 71, 13.2%) (Fig. 1). The median AI was 0.31 (IQR: 0.15, 95% CI: 0.2938–0.3120) for TD, 0.53 (IQR: 0.02, 95% CI: 0.5238–0.5344) for neurodivergent, and 0.69 (IQR: 0.24, 95% CI: 0.7267–1.0029) for ASD.

### Event-specific results

A total of 166 participants with complete classification and gender data were included in the analysis for the Arab Health, Dubai event. Based on AI scores, 142 participants (85.5%) were classified as TD, 10 (6.0%) as neurodivergent, and 14 (8.4%) as ASD. Median AI scores demonstrated a stepwise increase across groups, with 0.37 (IQR = 0.09) in the TD group, 0.53 (IQR = 0.02) in the neurodivergent group, and 0.61 (IQR = 0.06) in the ASD group. Among those classified as TD, 85 were male and 57 were female. The neurodivergent group included an equal number of males and females (*n* = 5 each), while the ASD group comprised 11 males and 3 females, resulting in a male-to-female ratio of approximately 3.7:1.

For the Web Summit, Qatar event, 202 participants were analyzed. Of these, 177 (87.6%) were classified as TD, 7 (3.5%) as neurodivergent, and 18 (8.9%) as ASD. The median AI score was 0.25 (IQR = 0.14) for TD, 0.53 (IQR = 0.00) for neurodivergent, and 0.70 (IQR = 0.21) for ASD. Gender distribution within the TD group included 103 females and 74 males. In the neurodivergent group, there were 2 females and 5 males, while the ASD group consisted of 6 females and 12 males, corresponding to a male-to-female ratio of 2:1 among those classified as ASD.

At the INSAR 2025 Annual Meeting event, Washington, USA event, 169 participants were included in the analysis. Based on AI scores, 125 participants (74.0%) were categorized as TD, 5 (3.0%) as neurodivergent, and 39 (23.1%) as ASD. Median AI scores were 0.27 (IQR = 0.15) for TD, 0.52 (IQR = 0.03) for neurodivergent, and 0.77 (IQR = 0.39) for ASD. The TD group comprised 82 females and 43 males, the neurodivergent group included 3 females and 2 males, and the ASD group consisted of 28 females and 11 males, yielding a male-to-female ratio of approximately 1:2.5 within the ASD group (Table 1).

**Table 1:** Summary of participant characteristics by event and classification group

Event	Group	Male (*n*)	Female (*n*)	Total (*n*, %)	Median AI (IQR)
Arab Health (*n* = 166)	TD	85	57	142 (85.5%)	0.37 (0.09)
	neurodivergent	5	5	10 (6.0%)	0.53 (0.02)
	ASD	11	3	14 (8.4%)	0.61 (0.06)
Web Summit Qatar (*n* = 202)	TD	74	103	177 (87.6%)	0.25 (0.14)
	neurodivergent	5	2	7 (3.5%)	0.53 (0.00)
	ASD	12	6	18 (8.9%)	0.70 (0.21)
INSAR Annual Meeting, USA (*n* = 169)	TD	43	82	125 (74.0%)	0.27 (0.15)
	neurodivergent	2	3	5 (3.0%)	0.52 (0.03)
	ASD	11	28	39 (23.1%)	0.77 (0.39)

Across the full sample, 210 males (80.8%) and 234 females (84.8%) were classified as TD, while 12 males (4.6%) and 10 females (3.6%) were classified as neurodivergent. Additionally, 38 males (14.6%) and 32 females (11.6%) were classified as having ASD, resulting in a male-to-female ratio of approximately 1.19:1 among those classified with ASD (Table 2).

**Table 2:** Gender distribution across AI-based classification groups

Group Classification	Male (*n* = 248)	Female (*n* = 289)	Total (*n* = 537)
Typically Developed (TD)	202 (81.5%)	242 (83.7%)	444 (82.8%)
Neurodivergent Group	12 (4.8%)	10 (3.5%)	22 (4.1%)
Autism Spectrum Disorder (ASD)	34 (13.7%)	37 (12.8%)	70 (13.1%)

### Group and gender comparisons

**Fig. 2:**
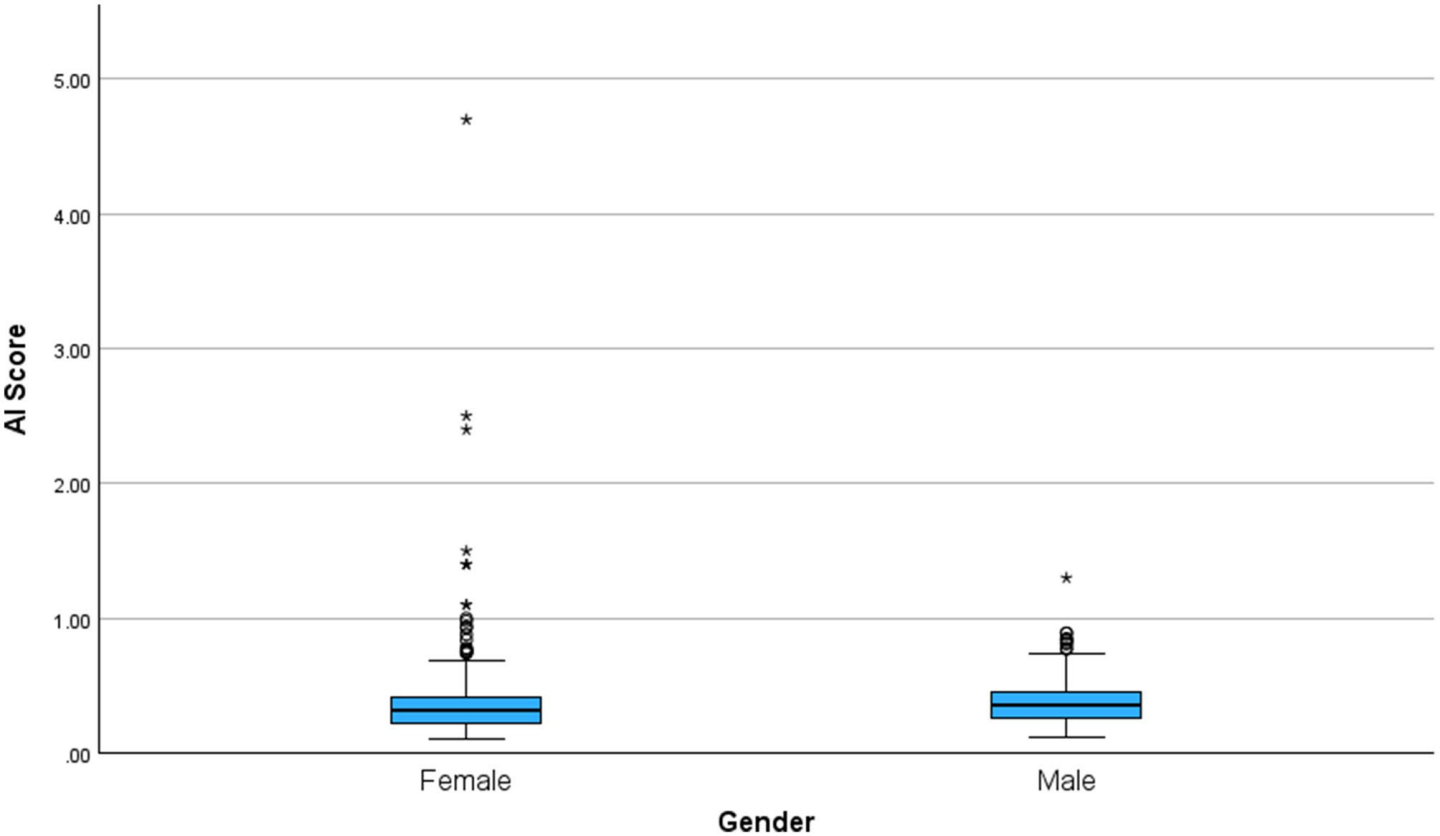
Box plot representing the distribution of AI scores across gender

Exploratory analyses revealed a statistically significant gender-based variation in AI scores, with males exhibiting significantly higher AI scores than females (Mann-Whitney U = 31,899, Z = − 2.197, *p* = 0.028). Figure 2 illustrates the distribution of AI scores by gender. The female group displayed a wider distribution (range = 4.59, SD = 0.376) with numerous high-value outliers, including a maximum AI score of 4.70. In contrast, the male group exhibited a narrower range (range = 1.18, SD = 0.170) and fewer extreme values, with a maximum score of 1.30.

The Kruskal-Wallis test revealed a statistically significant difference in AI scores among the TD, neurodivergent, and ASD groups (H(2)] = 231.987, *p* < 0.001). The highest mean AI score rank was observed in the ASD group (503.00), followed by the neurodivergent group (456.50), while the TD group had the lowest mean rank (223.00). Post-hoc pairwise comparisons using the Bonferroni correction showed that AI scores were significantly higher in the ASD group compared to the TD group (adjusted *p* < 0.001), and also significantly higher in the neurodivergent group compared to the TD group (adjusted *p* < 0.001). However, no significant difference was observed between the neurodivergent and ASD groups (adjusted *p* = 0.660).

**Fig. 3:**
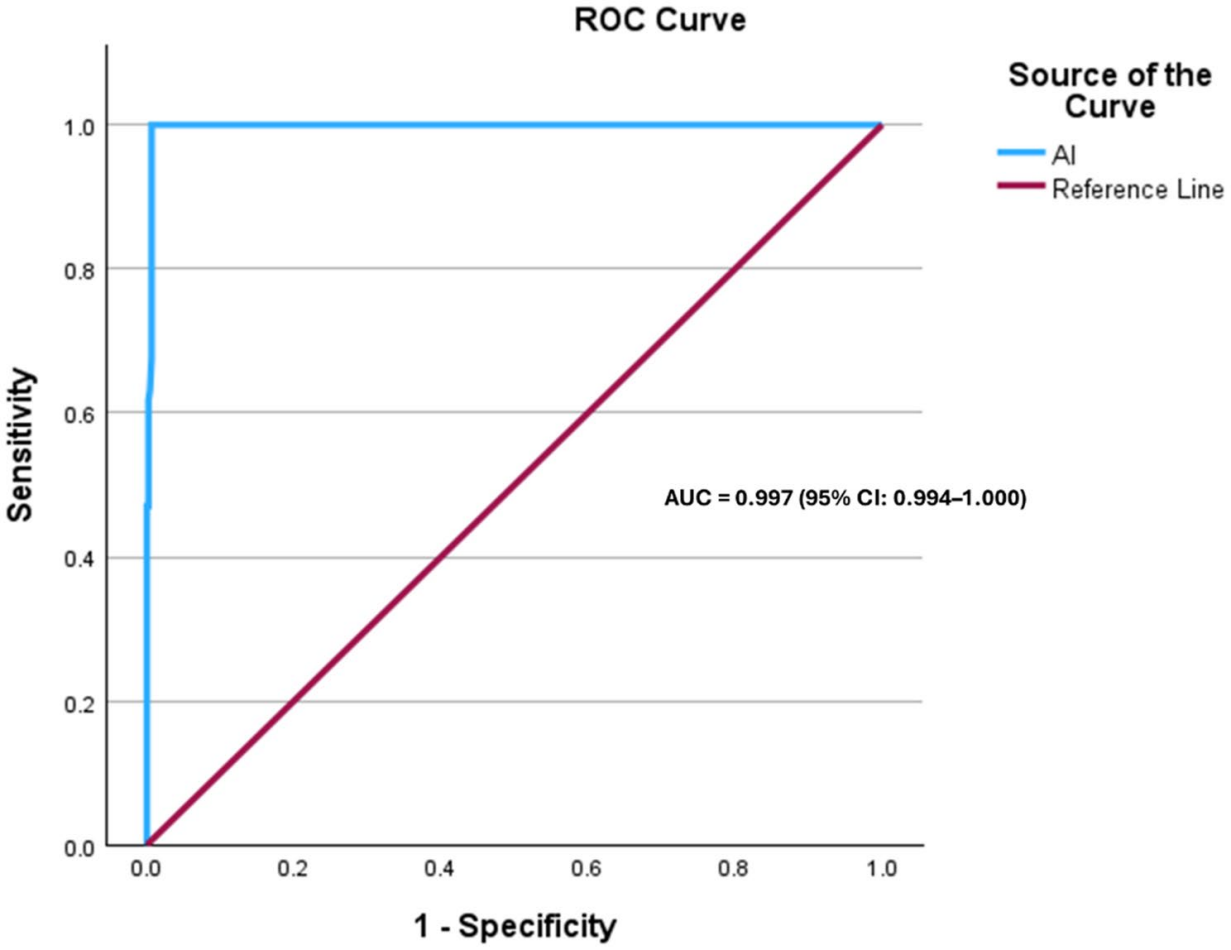
Receiver operating characteristic (ROC) curve for the Autism Index (AI)

The diagnostic performance of the AI was assessed using receiver operating characteristic (ROC) curve analysis (Fig. 3). The AI demonstrated outstanding discriminatory ability, with an area under the curve (AUC) of 0.997 (95% CI: 0.994–1.000, *p* < 0.001). The optimal cut-off score, determined using Youden’s Index, was ≥ 0.555, yielding a sensitivity of 100% and a specificity of 99.4%. The corresponding positive likelihood ratio (LR+) was 166.7, while the negative likelihood ratio (LR−) was 0.00.

## Discussion

In this study, we evaluated the real-world application of an Arabic and English-language eye-tracking stimuli paradigm for ASD screening and diagnosis within a general population setting. As anticipated, the ASD group showed higher AI scores compared to the TD group, consistent with previous research on social attention deficits in autism [4, 19]. These differences are in line with findings from eye-tracking studies where individuals with ASD tend to exhibit diminished social attention [8, 23]. The higher AI scores for the ASD group were particularly evident when compared to the TD group. These results align with prior research demonstrating that eye-tracking metrics can reliably differentiate ASD from non-ASD individuals [5, 17, 24, 25]. Notably, the highest proportion of ASD-positive participants was observed at the INSAR 2025 Annual Meeting event. This may be attributed to the nature of the conference itself, as prior research suggests that individuals with autistic traits are more likely to be represented in scientific fields [26]. However, no significant differences were found between the ASD and neurodivergent groups. This finding does not contradict prior validation findings [22]. In this heterogeneous, real-world sample, overlap in AI scores likely reduced separation between these groups. Although participants were classified into mutually exclusive categories by definition, the neurodivergent group may have included individuals with subthreshold or undiagnosed ASD traits, contributing to overlapping score distributions. Prior work has shown substantial overlap in social and cognitive traits across autism and other neurodivergent conditions, supporting a dimensional rather than categorical interpretation of group differences [3, 20, 21, 27–32]. In terms of measurement consistency, the distribution of AI scores was skewed, suggesting the presence of outliers, particularly in the ASD group, as observed in the boxplots. These outliers are common in clinical populations and may represent extreme cases of autism severity, reflecting individual differences in social attention, a feature consistently observed in previous studies [5, 33].

Gender-based analyses suggested a trend toward higher AI scores in males, which is consistent with the overall sample where a greater proportion of males were classified as ASD. This aligns with well-established epidemiological data showing that ASD is approximately 3.8 times more prevalent in boys than in girls [34], as well as prior studies reporting higher AI scores in males compared to females. This is further supported by our findings, which indicate that males exhibit significantly higher AI scores than females (*p* = 0.028). The male-to-female ratios observed at the Arab Health and Web Summit events more closely reflect general population trends, where ASD is more commonly diagnosed in males. In contrast, the unusually high proportion of females classified as ASD at the INSAR event may be explained by the fact that the number of females (*n* = 113) was double that of males (*n* = 56). These observations align with existing literature highlighting gender differences in ASD prevalence and presentation [35]. Moreover, this trend is consistent with findings from Frazier et al. [3], who noted that social attention patterns may differ between males and females, with females generally exhibiting a stronger preference for social stimuli. These initial gender differences suggest that biological gender might influence gaze behaviors, warranting further investigation to confirm these findings in larger and more diverse cohorts.

Our findings further reinforce the utility of eye-tracking as an objective marker for social attention deficits, supporting previous research indicating that gaze-based metrics can be translated into autism risk indices with high discriminative power [33, 36]. The fact that 95% of participants classified as ASD reported a formal diagnosis supports the validity of the eye-tracking paradigm as a reliable screening tool for use in general population settings. The ROC analysis demonstrated strong discriminatory performance of the AI score in this real-world setting, yielding a high AUC and favorable sensitivity and specificity at the optimal cut-off. These findings suggest that the eye-tracking paradigm can reliably differentiate individuals with autism-related gaze patterns from those without. Although gold-standard diagnostic assessments can reliably identify ASD in many high-resource settings, these approaches are subjective, time-intensive, require specialized training and are not universally accessible [37, 38]. In contrast, eye-tracking offers a scalable, objective, and relatively low-burden approach that can complement and aid existing diagnostic pathways rather than replace them. Its portability and minimal reliance on specialist expertise make it particularly well suited for large-scale screening, community-based applications, and use in low-resource settings where access to specialized diagnostic services is limited [39]. In such contexts, eye-tracking–based screening may help prioritize referrals, reduce diagnostic delays, and identify individuals who might otherwise remain undiagnosed. Notably, a small number of participants who screened positive based on their AI scores reported no prior diagnosis but indicated that they had previously been advised by a physician to seek further evaluation, suggesting potential diagnostic oversight or barriers to follow-up. This observation highlights the potential of the tool to identify individuals who may otherwise remain unrecognized by current diagnostic pathways. The results also suggest potential cross-cultural applicability, as similar trends have been observed in Western and non-Western populations [3]. A key strength of this study is the application of an eye-tracking paradigm in a large, real-world, non-research setting. Unlike traditional ASD diagnostic studies conducted in controlled laboratory environments, our findings were derived from a general adult population attending major international conferences. This setting provided a unique opportunity to assess the usability, feasibility, and engagement of eye-tracking technology as a potential public screening and diagnostic tool. Building on the established clinical validity of the paradigm, future studies may extend these findings by examining performance in larger, independently recruited cohorts.

### Limitations

Several limitations should be considered when interpreting these findings. Autism classification in this study was based on self-reported prior diagnosis rather than formal clinical evaluation. While this reflects real-world reporting and is common in population-based studies, it may have influenced diagnostic performance estimates, particularly in individuals with undiagnosed ASD. Although participants were asked about prior ASD diagnosis and any previous clinical concern or referral, diagnostic status was not confirmed using standardized assessments; therefore, undiagnosed ASD may have been present among participants classified as neurodivergent or typically developing, potentially influencing ROC performance. In addition, comorbid conditions were not systematically assessed in this non-clinical setting and may have contributed to variability in gaze patterns. Finally, demographic and cognitive variables, including age, years of education, and IQ, were not systematically collected; as these factors can influence gaze behavior and eye-tracking measures, their absence represents an additional limitation of the study.

## Conclusion

In conclusion, our results contribute to the growing evidence supporting eye-tracking technology as a valuable tool for ASD diagnosis. This paper demonstrates the feasibility of using an eye-tracking paradigm in a public setting to screen for ASD. The results support previous findings on gaze-based differences in ASD and highlight the potential for eye-tracking technology to serve as an accessible, objective screening and diagnostic tool in diverse populations. As we continue to develop objective and reliable biomarkers for ASD, the integration of such tools in clinical practice has the potential to improve early diagnosis and intervention outcomes, ultimately enhancing our understanding of the disorder across diverse populations. Future research should focus on exploring its potential integration into large-scale screening programs.

## Data Availability

The datasets generated and/or analyzed during the current study are not publicly available due to restrictions related to participant privacy and confidentiality but are available from the corresponding author on reasonable request.

## Abbreviations

ASD: Autism Spectrum Disorder
AI: Autism Index
TD: Typically Developing
ROC: Receiver Operating Characteristic
AUC: Area Under the Curve
IQR: Interquartile Range
LR+: Positive Likelihood Ratio
LR−: Negative Likelihood Ratio
